# Engineering a mobile health tool for resource-poor settings to assess and manage cardiovascular disease risk: SMARThealth study

**DOI:** 10.1186/s12911-015-0148-4

**Published:** 2015-04-29

**Authors:** Arvind Raghu, Devarsetty Praveen, David Peiris, Lionel Tarassenko, Gari Clifford

**Affiliations:** Institute of Biomedical Engineering, Department of Engineering Science, Old Road Campus Research Building, Headington, OX3 7DQ Oxford UK; The George Institute for Global Health, Inner Ring Rd Mada Manzil, Banjara Hills, Hyderabad, 500004 India; The George Institute for Global Health, Level 13, 321 Kent Street, 2000 Sydney, Australia; The University of Sydney, NSW, 2006 Sydney, Australia; Emory University, 201 Dowman Dr, 30322 Atlanta, USA; Georgia Institute of Technology, North Ave NW, 30332 Atlanta, USA

**Keywords:** Mobile health, Cardiovascular disease risk, Clinical decision support tool, Primary care, Chronic disease, LMIC, India

## Abstract

**Background:**

The incidence of chronic diseases in low- and middle-income countries is rapidly increasing both in urban and rural regions. A major challenge for health systems globally is to develop innovative solutions for the prevention and control of these diseases. This paper discusses the development and pilot testing of SMARTHealth, a mobile-based, point-of-care Clinical Decision Support (CDS) tool to assess and manage cardiovascular disease (CVD) risk in resource-constrained settings. Through pilot testing, the preliminary acceptability, utility, and efficiency of the CDS tool was obtained.

**Methods:**

The CDS tool was part of an mHealth system comprising a mobile application that consisted of an evidence-based risk prediction and management algorithm, and a server-side electronic medical record system. Through an agile development process and user-centred design approach, key features of the mobile application that fitted the requirements of the end users and environment were obtained. A comprehensive analytics framework facilitated a data-driven approach to investigate four areas, namely, system efficiency, end-user variability, manual data entry errors, and usefulness of point-of-care management recommendations to the healthcare worker. A four-point Likert scale was used at the end of every risk assessment to gauge ease-of-use of the system.

**Results:**

The system was field-tested with eleven village healthcare workers and three Primary Health Centre doctors, who screened a total of 292 adults aged 40 years and above. 34% of participants screened by health workers were identified by the CDS tool to be high CVD risk and referred to a doctor. In-depth analysis of user interactions found the CDS tool feasible for use and easily integrable into the workflow of healthcare workers. Following completion of the pilot, further technical enhancements were implemented to improve uptake of the mHealth platform. It will then be evaluated for effectiveness and cost-effectiveness in a cluster randomized controlled trial involving 54 southern Indian villages and over 16000 individuals at high CVD risk.

**Conclusions:**

An evidence-based CVD risk prediction and management tool was used to develop an mHealth platform in rural India for CVD screening and management with proper engagement of health care providers and local communities. With over a third of screened participants being high risk, there is a need to demonstrate the clinical impact of the mHealth platform so that it could contribute to improved CVD detection in high risk low resource settings.

## Background

Diseases that may affect the heart, arteries and circulation of the blood are collectively referred to as Cardiovascular disease (CVD). This can include ailments such as stroke, coronary heart disease, atherosclerosis, or peripheral arterial disease [1] and account for the majority of deaths worldwide according to the World Health Organization (WHO) [2]. Over 80% of these deaths occur in Low- and Middle-Income Countries (LMICs) [3]. In developing countries like India, even though approximately 70% of the total population live in rural areas, the doctor to patient ratio is lesser in rural areas (1:20000) as compared to the urban areas (1:2000) [4]. Access to prevention and control of CVD at the primary care level is poor in these communities. Even the use of medications for secondary prevention of stroke or myocardial infarction is very low [5]. Furthermore, the epidemiological transition from communicable to chronic disease as the primary cause of mortality has posed a substantial challenge to the health systems of LMICs. There is a need to prioritise limited resources to manage the exponential demands of the health system [6]. The existing physician-centric, western style care is therefore unsustainable in these regions and there is a need for more innovative solutions. One such solution is to expand the capacity of the existing healthcare workers and provide them with appropriate tools to manage CVD [7]. In 2005, the National Rural Health Mission (NRHM) was launched by the Indian Government with the aim of providing efficient primary health infrastructure for its vast rural population. One of the key features in this framework was the creation of the Accredited Social Healthcare Activists (ASHAs) per 1000 population, who are female healthcare workers acting as an interface between the people in rural areas and their public health centre.

With 5.4 billion mobile phone subscriptions just in the developing world today [8], the penetration of mobile phones has been substantial and continues to grow. This is an enabler for the delivery of healthcare services through mobile technology. Mobile health (or mHealth) has therefore been the subject of considerable research in recent times with a vast number of studies aimed at exploring its potential. The impact of mHealth on community health workers in LMIC was reviewed by Källander et al. [9], who reported the common mHealth interventions in the literature to be text messages and phone reminders to encourage healthy behaviour, attendance at follow-up appointments, and healthcare data collection. The authors also noted the need for innovation and research towards mHealth solutions for clinical decision support. Beratarrechea et al. [10] reviewed mHealth interventions on chronic disease in LMICs and found a positive impact on disease outcomes even though the number and size of studies were small. A recent review on mHealth for LMICs has concluded that despite the potential of a variety of applications in non-communicable disease care, mHealth is currently dominated by only behaviour change interventions [11].

There is substantial literature discussing CVD risk prediction and well-cited articles and reviews [12-14] have mentioned the need to focus research on the usage of risk algorithms in clinical practice for decision making, patient motivation, communication of absolute risk as well as understanding physician approval, behaviour and usage. At the same time, limited evidence exists in literature on using electronic CDS systems for CVD risk in resource-constrained environments. Other studies in LMICs, such as that by Ali et al. [15] who reviewed electronic CDS tools for diabetes care, highlight the need for well-designed investigations on the feasibility of using technology in clinical decision support.

In this study, we have designed and pilot tested a multifaceted healthcare worker intervention utilising an mHealth platform that comprises a clinical decision support tool for CVD risk assessment and management in rural India. The outcomes focussed on a preliminary evaluation of the tool for utility, effectiveness and acceptability by the ASHAs and community participants in this setting in order to inform large-scale evaluation.

## Methods

### Data collection

Data on CVD and associated risk factors were collected through an mHealth system consisting primarily of a client side mobile application and server side electronic medical record. The mobile application was compartmentalised into four steps, which enabled health workers to conveniently assess rural participants for CVD risk and disseminate appropriate recommendations. Table 1 describes the data acquisition process through the mobile application.

**Table 1:** **Table showing the 4 step process designed in the CDS tool for collecting patient data and decision support**

**Step 1**	**Step 2**	**Step 3**	**Step 4**
**(Demographics)**	**(Medical history)**	**(Risk factor acquisition)**	**(Decision support)**
Age	*Past history* of Myocardial infarction/Angina	Blood Pressure	*Recommendations*
Gender	Past history of Stroke	Blood Glucose	(Smoking cessation,
Location	Past history of PVD	(fasting/random)	nutrition and
Patient ID	Past history of Diabetes	Cholesterol	lifestyle modifications),
Name	*Family history* of Myocardial infarction/Angina	(TC,HDL,LDL,TG)	*Next Visit*
	Family history of Stroke	Height	(CVD risk screening,
	Family history of Diabetes	Weight	Diabetes screening
			Doctor referral),
			*Medication*
			(Blood pressure lowering,
			Lipid lowering,
			Anti-platelet therapy),
			*Tagets*
			(SBP, DBP)

Terminology used: PVD - Peripheral Vascular Disease, SBP - Systolic Blood Pressure, DBP - Diastolic Blood Pressure, TC - Total Cholesterol, LDL - Low Density Lipoprotein, HDL - High Density Lipoprotein, TG - Triglycerides, Patient ID - Patient Identifier.

### Computing 10-year absolute CVD risk

The World Health Organisation/International Society of Hypertension (WHO/ISH) provide colour-coded charts [16] which were used for the prediction of a 10-year risk of fatal or non-fatal cardiovascular event (myocardial infarction or stroke) in different epidemiological sub-regions of the world. Depending on the availability of cholesterol information, Low Information (LI) or High Information (HI) versions of the CVD risk charts can be used. The colour-coded ranges in the WHO risk charts indicated five levels of CVD risk for different values of risk factors [16]. The following information was necessary for the risk to be estimated:

- Presence or absence of diabetes
- Gender
- Smoking status
- Age
- Systolic blood pressure (SBP)
- Total blood cholesterol (TC), if known.

Treatment and recommendations for the management of CVD were based on Indian national and international guidelines. The prediction and management applications were stringently validated in two stages. The first stage was code validation, where the algorithm was coded by a physician in SPSS, and by an engineer as an Android application. This was run on a large external dataset from the Andhra Pradesh Rural Health Initiative [17] (which is a cross-sectional study of CVD risk-factors from people in rural Andhra Pradesh). The results were compared to find differences and iterative refinements were made to the algorithm and code to ensure full agreement. In the second stage, the same dataset was used where 100 individuals were randomly selected by a physician not involved in the algorithm development. The physician performed a manual assessment of CVD risk and treatment options, and compared her results with the assessments made by the CDSS on the same data. This was useful to refine the management options for CVD, which is outlined in the next section (Section ‘Estimating sub-conditions for management’ and ‘Management and treatment of CVD’).

### Clinical decision support

#### Estimating sub-conditions for management

Subsequent to the computation of a 10-year CVD risk for a given set of risk factors, the mobile application offered support for management of the participant’s risk and further follow-ups. In order to arrive at appropriate recommendations, the tool calculated sub-conditions (as listed in Table 2) that indicated high risk conditions or the presence of elevated glucose levels (impaired fasting glucose or IFG), high BMI (indicating obesity), or hypertension.

**Table 2:** **Computation of essential sub-conditions based on patient’s assessment**

**Clinically high risk**	**Impaired fasting glucose**	**Weight**	**Hypertension**
SBP ≥160 mmHg or DBP ≥100 mmHg or TC ≥320 mg/dL or LDL ≥240 mg/dL or TC/HDL>8	Fasting blood sugar level between 110 mg/dL and 126 mg/dL or history of diabetes	Obesity if BMI ≥30 Overweight if 25 ≤BMI<30	SBP ≥140 or DBP ≥90 or history of hypertension

This is performed as a precursor to CDS recommendations for CVD management; Terminology - BMI stands for Body Mass Index.

#### Management and treatment of CVD

After computation of the CVD risk score and sub-conditions for assessing high risk, the CDS tool was designed to offer point-of-care (POC) decision support to both ASHAs and physicians for the management of CVD. This included three categories - general recommendations, referral, and treatment recommendations and were based on international and national guidelines [18,19]. General recommendations are intended for controlling or modifying behavioural risk factors including smoking, alcohol consumption, physical inactivity, and diet/nutrition. Except detailed advice on smoking cessation (which are given only to smokers), other recommendations are advised to all users. A referral indicated the need for consulting a primary care physician, which was also the outcome for ‘next visit’ flags for absolute risk and diabetes screening. Treatment recommendations were available only to physicians. The CDS output for different CVD risk profiles are summarised under Table 3.

**Table 3:** **Table showing referral and medication recommendations for a particular CVD risk category**

**Risk category**		**Referral**		**Medication**
		**CVD risk screening**	**Doctor referral**	
High risk	≥30% risk	every 3-6 months	Yes	BP lowering therapy
	or past CVD history			Lipid lowering
				Antiplatelets
Intermediate	20 to <30% risk	every year	Yes	*BP lowering therapy*
risk				(if BP ≥ 140/90)
				*Lipid lowering*
				(if Diabetes is present
				or SBP ≥160 mmHg
				or TC>200 mg/dL or
				LDL>120 mg/dL)
	10 to <20% risk	every 2 years	Yes if	*BP lowering therapy*
			(Diabetes or	(if Diabetes is present
			IFG is present)	and BP ≥140/90
				or SBP ≥160 mmHg)
			or	*Lipid lowering*
			(history of diabetes	(if Diabetes is present
			and SBP ≥160 mmHg)	or SBP ≥160 mmHg)
Low risk	0 to <10% risk	every 5 years	Yes if	*BP lowering therapy*
			(Diabetes or	(if Diabetes is present
			IFG is present)	and BP ≥140/90
				or SBP ≥160 mmHg)
			or	*Lipid lowering*
			(history of diabetes	(if Diabetes is present
			and SBP ≥160 mmHg)	or SBP ≥160 mmHg)

### CDS tool design

An important factor in the success of any technology-based intervention depends on how well it can fit within the needs of the users and the environment [20]. In our context of managing CVD in resource-constrained settings, the challenge and opportunity was to design an appropriate tool that could be easily used by minimally trained health workers (who were the end users) as well as fit the needs of local primary care physicians and community participants.

Conventional methods of design and development relied on traditional “requirements engineering” [21], where the emphasis is on preparing thorough documentation with clear specifications prior to the development of a proposed solution. This is often the first phase in the waterfall software development model which relies on a pre-planned sequential design procedure [22]. Another popular practice in software engineering is the agile development methodology which emphasises iterative development with constant feedback from the stakeholders whereby requirements emerge along the process [23]. This has the advantage of evolving to changes in a dynamic environment.

**Figure 1:**
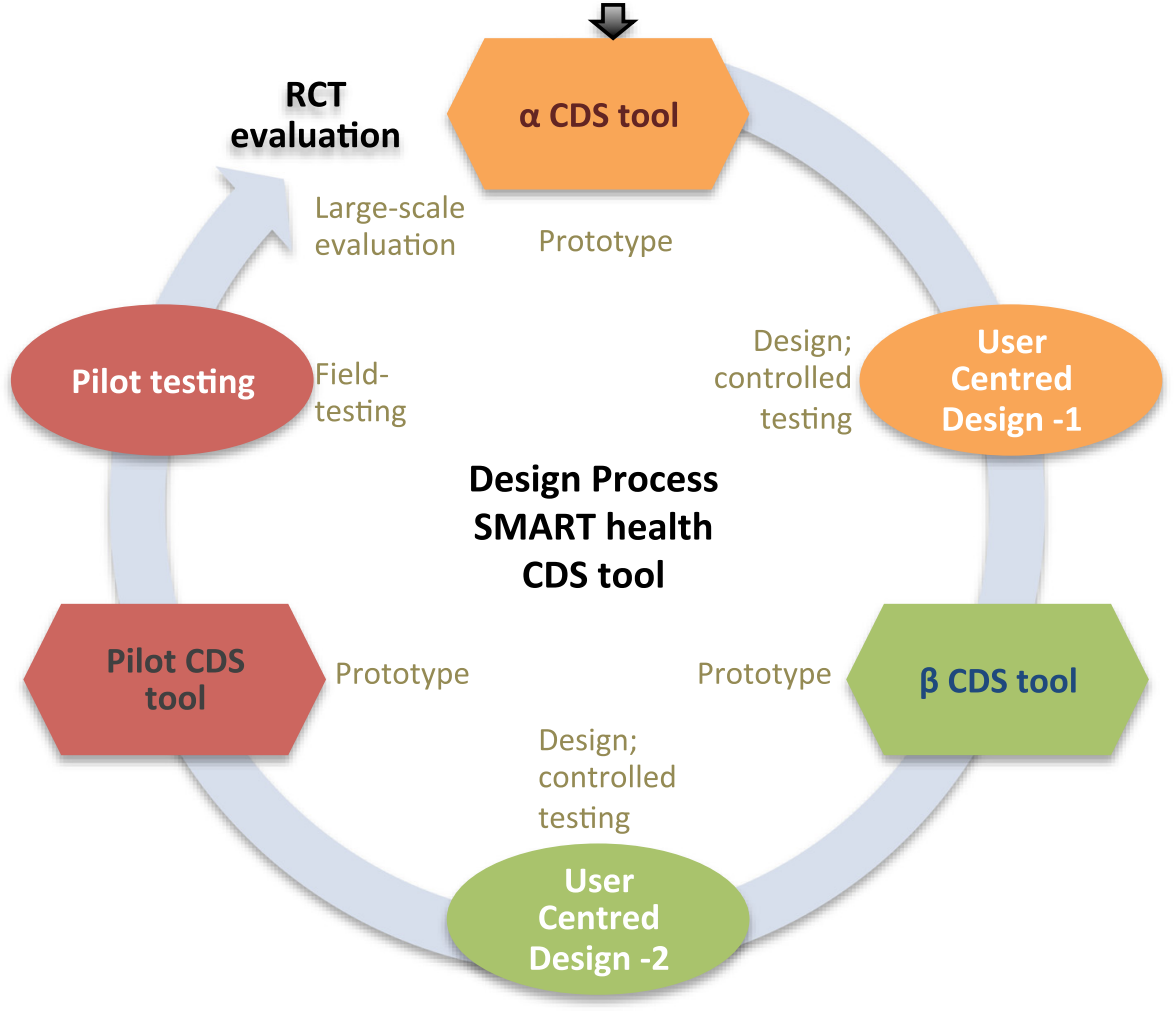
Phases of design and development following a User centred design aproach, where the aim was to bring out issues centred around the end user’s interaction with a prototype without placing any explicit demands on them. The process diagram shows prototypes in the form of an alpha (*α*) and beta (*β*) CDS tool, each followed by a user centred design phase. This led to the development of a tool for the pilot study, which upon further improvement would be suitable for a randomised controlled trial.

The requirements of our end-users compounded with the uncertainties in technical infrastructure (such as the availability of uninterrupted 2G/3G internet connectivity) could not be gauged thoroughly through a needs assessment. Therefore, the agile development approach was followed in our study to iteratively design prototypes and elicit feedback from the end-users. However, our approach differed from conventional practice since the end users had little or no experience with using information and communication technology and could not drive the design. To overcome this, firstly we employed a multidisciplinary team comprising an engineer, local physician, sociologist, and an expert physician to balance *system requirements* (for designing features for an effective intervention, such as understanding the local clinical practice for ways of conveying recommendations for managing CVD risk, or assessing accuracy of ASHAs asking questions like medical history) with *user requirements* (for instance, to observe if the ASHAs can compute the year of birth based on age). Secondly, we performed phases of prototyping and user assessment (which is illustrated through Figure 1) where the multidisciplinary team evaluated the end user interactions with the mobile application through observations and post-procedure interviews.

**Figure 2:**
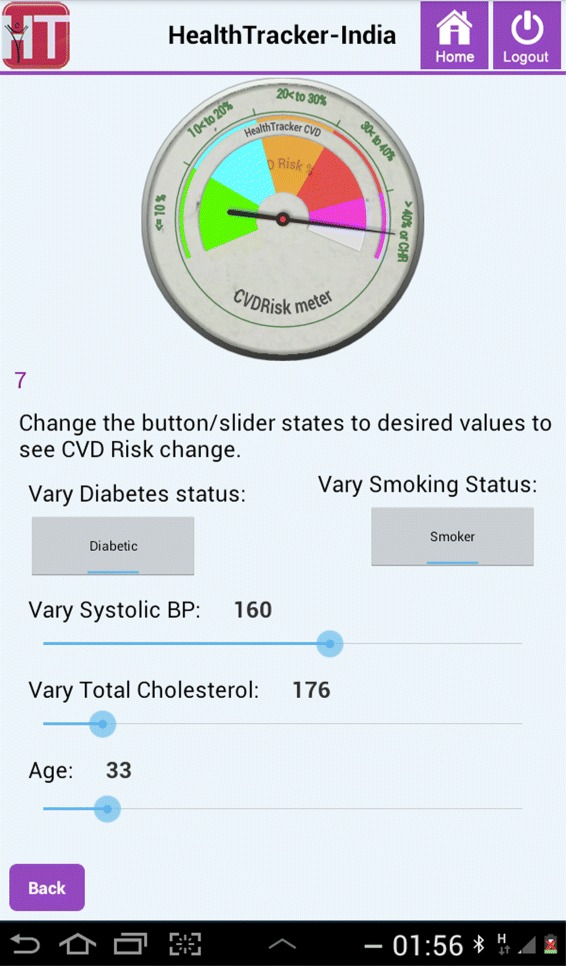
CVD Risk projection meter indicating present risk factor levels for the patient.

**Figure 3:**
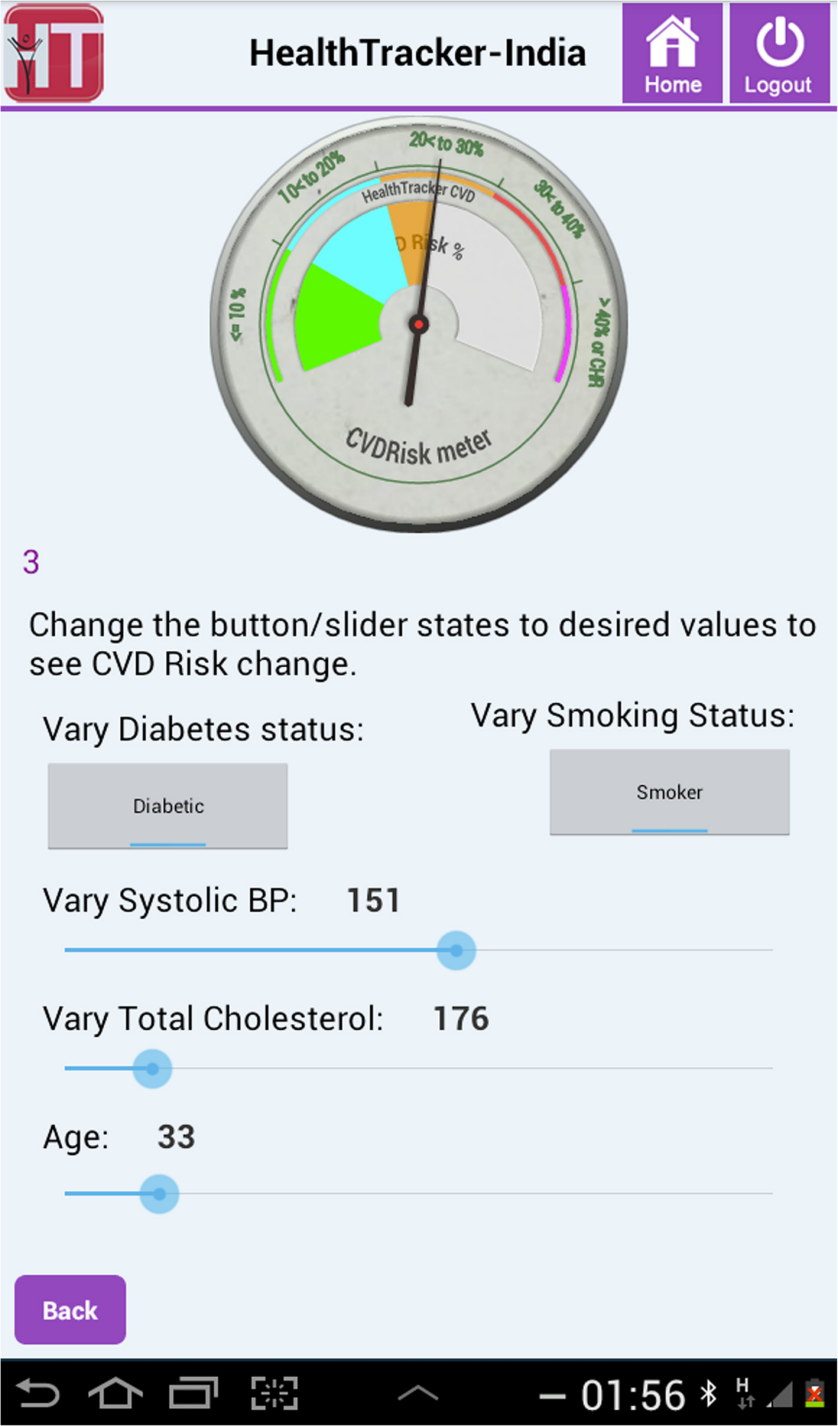
The projected 10-year risk if the patient reduces his blood pressure by approximately 10 mmHg, as shown by the CVD Risk projection meter.

Some of the key features that were incorporated through our agile approach included:

- **One touch navigation**
  Since the end user base had little or no experience, touch errors were common and frequent. The mobile application’s design was such that ASHAs could navigate through the majority of the content using a single button. Care was taken to ensure idiosyncrasies of the mobile-based device that interfered with workflow were minimal. For example, in certain cases the virtual keyboard was minimised subsequent to user input.
- **CVD Risk Projection meter**
  The application had a visual risk projection meter (as shown in Figures 2 and 3) to convey the meaning of CVD risk to the patient in an understandable way. For example, Figure 2 illustrates the case of a 33-year old smoker with diabetes. The participant has a blood pressure value of 160/89 mmHg, total cholesterol of 176 mg/dL and his risk of developing CVD over a 10-year period is shown to be high. If the participant reduced his blood pressure alone by approximately 10 mmHg (as shown in Figure 3), his 10-year risk of developing CVD is reduced substantially from above 40% to between 20% and 30%. Thus the cause and effect of each risk factor for a particular patient can be visually emphasised. This is intended to help the patients visualise the effect of controlling risk factors and was designed to encourage adherence to medication and a change in behaviour of modifiable risk factors, such as smoking.
- **Event calendar/age entry**
  It was found that some of the participants did not know their date or even year of birth accurately. This was due to factors such as date of birth being mandatory for registration only after the Indian Registration of Births and Deaths Act, 1969 [24]. Therefore, the mobile application used methods to increase the accuracy of the estimated age; a vital parameter in the risk assessment algorithm. A comprehensive list of well-known historical events (for example, Indian Independence day, 1947) could be retrieved in order to help the patient decide how old they were at that point in time. Also, for female patients, specific questions (mostly related to maternal health) that had a high probability of narrowing down the exact age were used.
- **Information buttons**
  These buttons were embedded in the standard risk assessment procedure and intended to disseminate essential information for a particular question the user wanted to know more about. For instance, the button could be used to recap the protocol for blood pressure measurement or retrieve a list of commonly used drugs to see if the patient had a history of taking any of them.
- **Review patient**
  The review patient feature offered the option to review existing patients who have been screened previously by that user. This was especially motivated by the recommendations of a physician who intended to use a staff nurse to screen patients before they had an appointment with him. Using the Review Patient feature, the physician could assess and review the participant’s CVD risk in an efficient way, thereby promoting greater ease of adoption of the tool in the primary care setting.
- **Accuracy of data entry**
  To ensure correctness of data entry and avoid transcription errors, on-screen checks were performed at the end of each step if the entered data was not clinically valid. Furthermore, a validated Blood Pressure (BP) device (Stabil-O-Graph [25]) was used, which could interface with the tablet via Bluetooth and send three BP measurements to the tablet. However, blood glucose was measured using the OneTouch Ultra2 glucometer which did not have Bluetooth functionality although it was desirable. This was because the recurring cost of test strips was prohibitively high for a Bluetooth-enabled glucometer. All data in the CDS tool could be corrected or inputted manually.

The CDS-based CVD risk assessment and management tool was built for devices running Android 4.0 and above, and supported both Telugu (local language) and English. The application was optimised to run on a higher-end version of a low-cost tablet (£100) for use by ASHAs, and a Samsung Galaxy 7-inch tablet (£160) to be used by physicians. Low-cost tablets were preferred for two reasons. The first was to assess if they could perform adequately in our setting so as to inform cost-effectiveness of the intervention. The second reason was the high healthcare-worker:doctor ratio in resource-constrained regions which required cost-effective tablets (since they can be replaced more easily if damaged or stolen).

### Field testing

The mobile based CDS tool was field tested by 11 ASHAs who performed door-to-door screening of community participants in 3 villages in rural Andhra Pradesh, India. Each ASHA was to screen approximately 20 participants in one month for high risk and a convenience sampling method was followed, where participants could be selected based on their accessibility and proximity to the ASHAs. The ASHAs were trained for one week to use the CDS tool and tablet. The data from each participant screened by the ASHA were uploaded to a secure server hosting an electronic medical record system (OpenMRS [26,27]). Decision support on treatment was available to physicians, who used the CDS tool as a standalone system to screen and manage patients visiting their clinic. The entire mHealth infrastructure was designed to be interoperable and leveraged the Sana Mobile platform, an open-source telemedicine framework [28,29].

A four point Likert scale [30] was used to evaluate usability at the end of every risk assessment performed by the ASHAs. To understand user behaviour and pattern of interaction with the mobile application, an analytics framework was built within the tool. It recorded the data and timestamp for every click made by the ASHAs as they performed CVD risk assessments. For ease of comparability of usage patterns and to highlight diversity in trends amongst different end-users, three distinct ASHAs were chosen based on the total number of assessments they performed and years of experience they had in performing their role. Everyone amongst the chosen three ASHAs were identified through interviews as being primarily a Telugu speaker, and claimed to be able to read and write English at secondary school level.

The variability in end-user usage was analysed in this study to gauge the performance of ASHAs. This was done in order to identify the stage at which additional support or training was needed if necessary. Data from the three ASHAs, representing all the ASHAs in performance, were analysed individually to obtain an estimate of their mean procedure time over the course of the pilot study. Bootstrapping was performed considering all samples available up until and including that procedure. This was performed to investigate bias towards uneven sampling. For example, if we are to estimate the mean completion time and confidence interval (CI) for an ASHA who has performed 10 risk assessments, her procedure times until and on the 10^th^ procedure would be taken as our sample for bootstrapping. The 95% CI for the mean was subsequently estimated and plotted individually for the three ASHAs. This is useful to understand how variable the ASHA’s own procedure times can be and with sufficient samples (of risk assessment procedures), we can reliably estimate how long an ASHA can take and how much training is needed until she is proficient to use the CDS tool (which may be shown by a narrow confidence interval).

The ASHAs manually entered the glucose values into the tablet, a process susceptible to errors. However, to assess the extent of errors, the blood glucose values stored in the glucometers were downloaded and compared with those values entered by the ASHA in the tablet during risk assessment. Care was taken to ensure that the glucose readings being compared were taken at the same time. This was performed by obtaining timestamps from the analytics framework in the CDS tool and the glucometer’s memory.

In-depth qualitative interviews with the ASHAs and focus group discussions were also undertaken to identify barriers to the adoption of mobile technology. These are detailed in the article by Raghu et al. [31] and are not presented in this paper.

Ethics: The study was approved by the ethics committee of Centre for Chronic Disease and Control, India and the University of Sydney, Austrlia (registration: CTRI/2013/06/003753). Informed, written consent was obtained from all participants contributing data in the study.

## Results

A total of 227 individuals (mean age of 51.4 ±13.1 years) were screened by 11 ASHAs, while 3 PHC physicians independently used the mobile application (*HealthTracker*) as a clinical decision support tool to screen 65 in-patients (mean age of 55.3 ± 11.7 years) who visited their clinic (total N=292). Although physicians used the mobile application, the results presented in this paper focuses entirely on usage by the ASHAs.

The three distinct ASHAs chosen for detailed usage analysis had a mean age of 29 ± 2 years and had the experience of using a mobile phone at the time of this study. We shall refer to the three ASHAs as *npB1*, *npM1*, and *npL1* and they had 3, 7, and 4 years of experience working as an ASHA and performed 28 (highest number in this study), 20 (median number of assessments in this study), and 16 (lowest number of assessments amongst ASHAs in our study) risk assessments respectively.

### CVD risk profile

**Figure 4:**
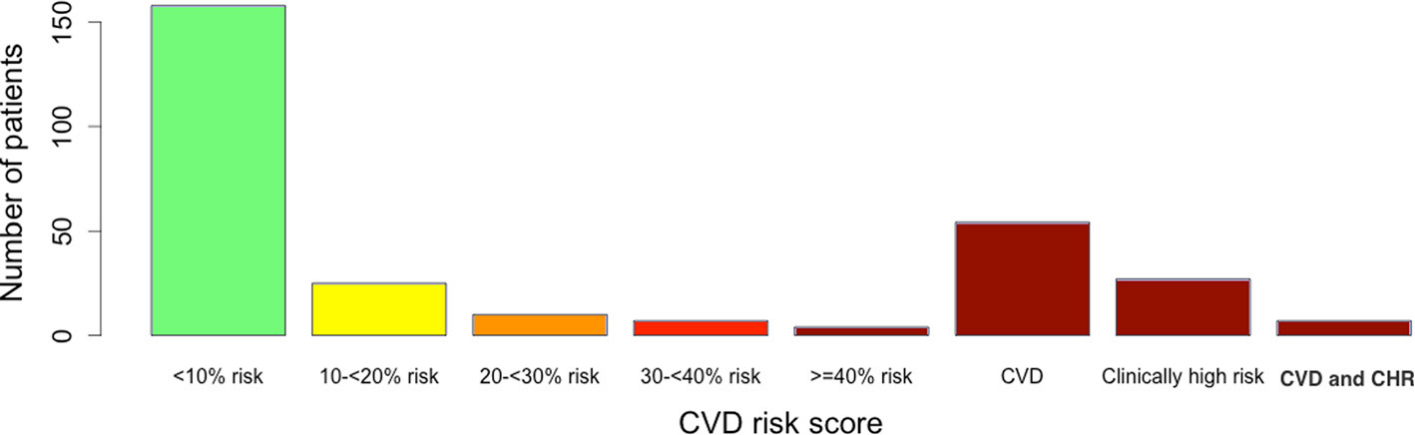
Distribution of CVD risk scores in the pilot population (N=292). CHR denotes clinically high risk. 34% of screened participants had a CVD risk score greater than 30% or had CHR or CVD or both.

Figure 4 shows the distribution of a 10-year risk of developing CVD for the participants for the study participants. Most individuals were either at low risk (<10%) or high risk (due to a previous incidence of CVD, or had a clinically high condition as previously defined in Table 2, or both).

### Evaluation of mHealth platform

#### Data Analytics -User behaviour and interactions

**Figure 5:**
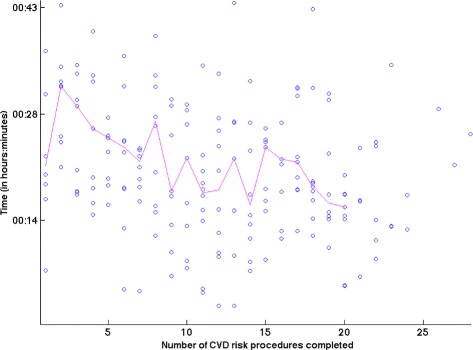
Graph illustrating the total CVD risk assessment procedure time over the number of procedures performed by the ASHAs. The median time for completion of a risk assessment was 00:21:10 (read as hours:minutes:seconds). The median time for all ASHAs was taken at every procedure and the resulting trend suggests a decrease in total procedure time as more CVD procedures were performed. The first procedure has a lower median time and this may be because it was performed immediately after the training phase for ASHAs.

**System Efficiency** The overall time taken for screening the community participant’s CVD risk over the duration of the study is shown in Figure 5. The median time for all ASHAs was 00:21:10 (read in terms of hours:minutes:seconds) with an IQR of 00:14:08. ASHAs *npB1*, *npM1*, and *npL1* took 00:27:28 (IQR 00:14:05), 00:24:20 (IQR 00:12:12), and 00:33:53 (IQR 00:32:41) respectively. A decreasing trend in completion time as users performed more procedures is observed.

**Figure 6:**
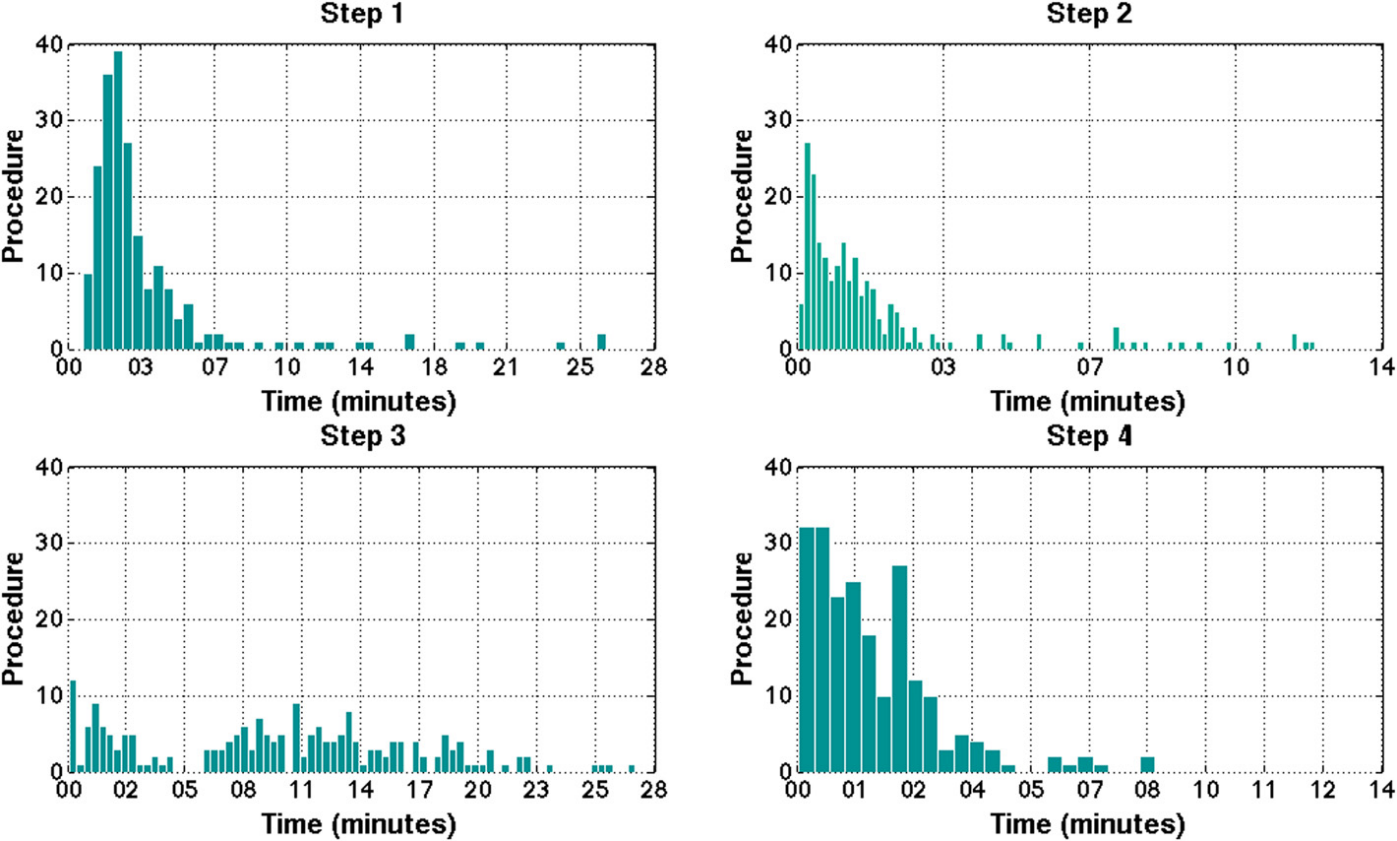
Assessment of individual step times, broken down as Step 1- Demographics with median time of 00:02:37 (IQR 00:02:16), Step 2- Medical History with median time of 00:01:08 (IQR 00:01:35), Step 3 - Risk factor acquisition with median time of 00:10:33 (IQR 00:11:22), and Step 4 - Decision support 00:01:30 (IQR 00:01:53).

The distribution of time taken for each step of CVD risk assessment for all ASHAs is shown in Figure 6. Step 3 (risk factor acquisition) took the longest during data collection and its extensive spread observed from the distribution shows a large variation (IQR 00:11:22) between ASHAs in completing that step.

**Figure 7:**
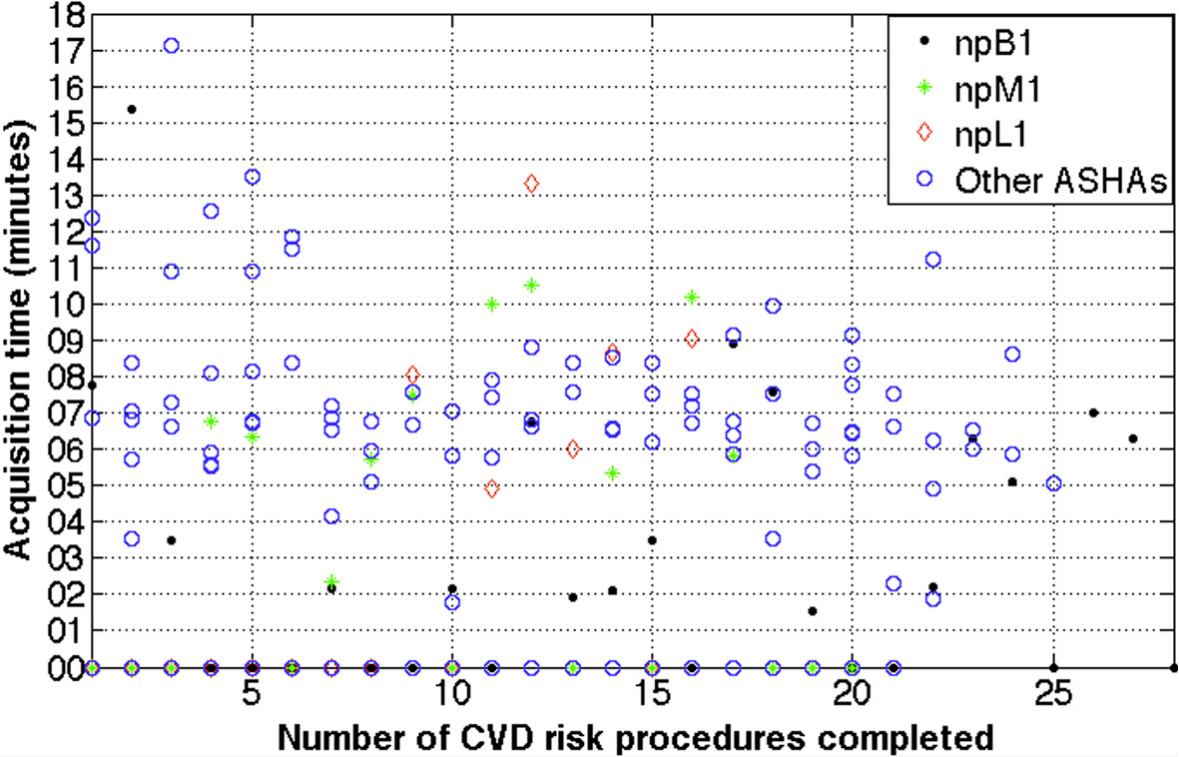
Plot of BP acquisition times over procedures performed on the course of our pilot study. Acquisition times at 0 indicate Bluetooth transmission was not attempted. Overall Bluetooth BP device usage rate was 55% and overall median acquisition time was 00:06:50 (IQR of 00:02:33). Four out of 219 procedures were not considered as the risk assessments were performed non-sequentially by the ASHAs.

The number of times the ASHA chose Bluetooth transmission over manual transmission (or the Bluetooth BP device usage rate) was analysed and is shown in Figure 7. Only one transmission from the BP device was needed for three BP measurements in each CVD risk assessment procedure. From Figure 7, ASHA *npB1* had a usage rate of 61% (17/28 procedures) with mean BP acquisition time of 00:05:20 (*σ* = 00:03:36) while *npM1* had a usage rate of 50% (10/28) with mean BP acquisition time of 00:07:06 (*σ* = 00:02:36). ASHA *npL1* had a usage rate of 37% (6/16) with mean BP acquisition time of 00:08:24 (*σ* = 00:02:56). The ASHA who used Bluetooth the most (*npB1*) for transferring BP readings had the lowest mean BP acquisition time. However, the ASHA with most experience (*npM1*) showed more consistency when acquiring BP.

Before the 10^th^ procedure, Bluetooth usage rate was 40% for *npB1*, 50% for *npM1*, and 10% for *npL1* while after the 10^th^ procedure (midway between their ‘assigned’ target of 20 procedures as previously described in Section ‘Field testing’), the usage rates were 65%, 44%, and 80% respectively.

**Figure 8:**
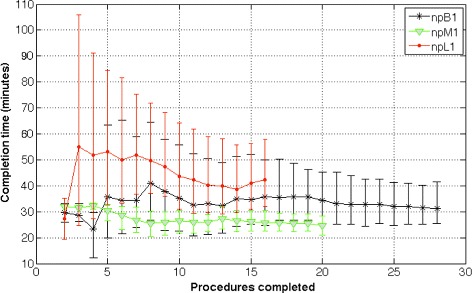
Estimate of the mean procedure times with 95% confidence intervals for ASHAs *npB1*, *npM1*, and *npL1* over successive procedures performed (for the duration of the pilot study). ASHA *npM1* has the narrowest CI and least estimated mean procedure time while *npL1* has the widest CI and highest estimated mean procedure time at end of the study. The estimate of 95% CI and mean procedure time was obtained using a bootstrapped sample that included risk assessments performed up until and including that time period in the study. This was performed to account for uneven sampling bias (for example, at the 5^th^ procedure, procedure 1 till 5 were taken as samples and bootstrapped). After ASHA *npL1*’s 13^th^ procedure, her estimated mean procedure time increases which suggests that she took much longer to complete the last two risk assessment procedures.

**User variability** From Figure 8, we observe that *npM1* is consistent and has the most narrow CI [00:21:01, 00:28:27] while *npL1* has the widest CI [00:31:58, 00:57:58] for the estimated mean procedure time towards the end of the study. The three ASHAs show less variability in procedure time over the course of the pilot study (when they complete more risk assessments).

In the initial stages of the pilot study (for instance, at the end of 5 procedures completed by each ASHA), the bootstrapped estimate of the mean procedure times for all ASHAs are high with wide CIs. ASHA *npL1* has the largest estimate with mean time 00:53:02, 95% CI [00:32:36, 01:24:35]. ASHAs *npM1* and *npB1* have comparable estimate of mean times but the latter has a much wider 95% CI (*npM1* - mean time 00:30:33, 95% CI [00:26:30, 00:32:45]; ASHA *npB1* - mean time 00:35:45, 95% CI [00:20:00, 01:03:28]).

At the end of 10 procedures (or approximately mid-way through the assigned target of 20 assessments for the ASHAs), the estimated mean time and CI continues to decrease (*npL1* - mean time 00:43:31, 95% CI [00:31:05, 01:04:19]; *npM1* - mean time 00:26:28, 95% CI [00:21:50,00:30:29]; *npB1* - mean time 00:35:18, 95% CI [00:24:34, 00:55:45]).

When 15 procedures have been completed by the ASHAs, the estimated mean stabilizes while CI continues to become narrower (*npL1* - mean time 00:41:05, 95% CI [00:30:56, 00:56:24]; *npM1* - mean time 00:26:18, 95% CI [00:22:34,00:30:22]; *npB1* - mean time 00:34:43, 95% CI [00:25:07, 00:52:08]).

At the end of the pilot study, *npL1* has an estimated mean time 00:42:15 with 95% CI [00:31:58, 00:57:58] that is compared to the last milestone (15 procedures) and there has not been an appreciable increase in the number of procedures the ASHA had performed. *npM1*, having completed 5 more procedures since the last milestone, finishes with a slightly lower estimated mean procedure time 00:24:42 with narrower 95% CI [00:21:01, 00:28:27]. *npB1* had finished 13 procedures more than the previous milestone and ends with a lower estimated mean time 00:31:21 with 95%CI [00:25:27, 00:41:32].

**Errors in manual entry of Blood Glucose measurements** Out of 227 patients assessed, 14 patients had an erroneous value of glucose level entered. The median error was 9.55 mg/dL with IQR 35.75 mg/dL (0.53 mmol/L with IQR 1.99 mmol/L).

**Usefulness of point-of-care management recommendations for ASHAs** Table 4 quantifies the extent to which the built-in management guidelines for CVD were used by the ASHAs through the number of clicks recorded in each management section (outlined previously in Table 1) for all procedures completed. It can be observed that the usage of the risk projection meter was much lower in comparison to the other sections for the three ASHAs. Also only 81% participants screened by ASHA npL1 were told about their next visit for follow-ups while in contrast, ASHA npB1 disseminated the information to 93% of her participants.

**Table 4:** **Statistics for how often the management recommendations in the CDS tool were actually used by the ASHAs**

**ASHA**	**Risk projection meter to communicate CVD risk association between risk factors**	**Recommendations (lifestyle, smoking, nutrition)**	**Next visit (doctor referral, CVD risk/diabetes screening)**
*npB1*	79% (22/28)	96% (27/28)	93% (26/28)
*npM1*	75% (15/20)	85% (17/20)	90% (18/20)
*npL1*	81% (13/16)	81% (13/16)	81% (13/16)

#### CVD referrals

An important component of the mobile based CDS tool is the referral indication (as mentioned in Section ‘Management and treatment of CVD’). Out of 227 participants screened by the ASHAs, the CDS tool identified 57% (n=128) for referral to a physician either for high CVD risk (n=88) or IFG (n=40).

#### Usability

The end users of the application were asked to complete a questionnaire at the end of every risk assessment procedure. In over 72% of the screening procedures performed, the mobile application was found easy to use for that particular procedure. Users concurred similarly on the usefulness of a graphic bar that visualised risk scores in communicating the meaning of absolute CVD risk reduction to the community participants. No user gave the application a rating below 3 on a scale of 4, with 4 being most useful and 1 being least useful. In less than 2% of the procedures performed, the ASHAs recorded difficulties with collection of risk factors (such as blood pressure, height and weight, blood glucose).

## Discussion

The CDS tool was designed and developed using the agile development methodology with the close engagement of the ASHAs and physicians. The process was repetitive until the ease of use was confirmed by the end-users and this stage became the final version of the CDS tool. A number of key features were added through this process. The use and integration of open-source telemedicine platforms such as OpenMRS and SanaMobile were intended to increase interoperability. This is important because in the area of mHealth application development, there exists silos of numerous non-interoperable mHealth applications which are likely to increase the risk of duplication in effort and fragmentation of purpose. The risk profiles of screened participants in the three villages in Andhra Pradesh had a bimodal risk distribution, where the participants were mostly either low risk or high risk. The number of participants at high risk of CVD was almost one third of the screened sample. There was a decrease in median completion time with more number of procedures performed. This indicated the ASHA’s increased familiarity with the process as well as provides evidence for expected or optimal usage and actual usage. The median procedure time for ASHAs was 21 minutes for a complete CVD risk assessment with Step-3 (risk factor acquisition) taking maximum time (of over 10 minutes although it recorded large variations between ASHAs). Acquisition of three BP measurements, on its own, took approximately 7 minutes. This could be useful to assess the maximum number of community participants that can be screened by an ASHA given her usual commitments towards antenatal care.

It is interesting to compare the usage patterns of the three ASHAs (*npB1*, *npM1*, and *npL1*) from four parameters that we have analysed - total number of risk assessments, median completion time and 95% CI, use of Bluetooth functionality, and rate of disseminating management recommendations to participants. *npB1* may be classified as one of the better performing ASHAs in the pilot study as, firstly, she performed the highest number of risk assessments amongst all ASHAs. Secondly, she had the highest usage rate of Bluetooth functionality and lowest mean BP acquisition time. Thirdly, she had the highest rate of disseminating management recommendations although her mean procedure time and 95% CI was fairly average compared to other ASHAs. *npM1* showed more consistency than any other ASHA in all aspects of her performance. The number of assessments she completed was exactly the same as the approximate target of 20 procedures prescribed for all ASHAs at the start of the pilot. With regard to the automated Bluetooth feature, the usage rate was almost the same before the ASHA’s 10^th^ procedure (50% usage rate) and after her 10^th^ procedure (44% usage rate). With the lowest estimated mean time and smallest 95% CI as well as an approximately average rate of disseminating management recommendations, ASHA *npM1* was the most consistent performer. *npL1* performed the fewest assessments, had the highest estimated mean time with a wide 95% CI, low dissemination rate of management recommendations, and the lowest usage rate of the automated Bluetooth feature. However, with regard to Bluetooth usage, we observe that even though only a single attempt (or 10%) was made before her 10^th^ procedure, her usage rate increased to 80% thereafter. Though this may indicate a longer learning curve, ASHA *npL1* may be identified as one that would need additional attention or performance monitoring. By recording user interactions with the mobile application, we could thus gauge the end-user’s behaviour and effectiveness of the tool’s features such as the rate of dissemination of point-of-care CVD management recommendations.

The introduction of automated Bluetooth BP measurements in this study found low adoption initially. However, we observed that towards the end of the study, the ASHAs recognised the ease of use and utility of this feature, as was observed through qualitative interviews (not reported in this paper). ASHAs also recorded difficulties with operation of the BP monitor. Given the current overall adoption rate of 55%, a less sophisticated BP monitor with faster acquisition times may increase adoption and optimise time taken for Step 3. The errors reported from manual data entry during blood glucose measurement demonstrate that transcription and transposition errors were likely and the erroneous values had a wide range. With regard to POC decision support, communication through the risk meter found the lowest adoption in Table 4 and this is possibly due to difficulties in the ASHA’s understanding of the risk projection feature. The overall user trends in user interactions was observed by the usage of our analytics framework in the CDS tool. This was beneficial to understand learnability [32], which is an indicator of how easily the ASHAs performed tasks as well as efficiency [33], which is an analysis of the ASHA’s ability to quickly perform the task they have learnt. The understanding of these parameters can aid in better design (for example, by evaluating features widely used versus those un-used or difficult to use) and learn about user performance over time so that adequate support may be given to those ASHAs who need technical assistance (for instance if the ASHA’s efficiency declines, as her estimate of mean procedure time increases successively).

By involving the end-users iteratively as well as acquiring information through data analytics, we minimise social desirability bias [34,35], an important issue in acceptance testing in global health. Another key aspect of our study is the design thinking process that has cohesively involved the local community participants and end-users, and therefore differs from global health projects which ’push’ solutions onto the community. The in-depth design strategies resulted in a tool that could be easily integrated within the workflow of the ASHAs, as reflected by the majority agreeing with its easy of use, usefulness, and increased speed of assessments over time.

The main limitation in this pilot study was the limited sample size. However, this was not designed to be representative. With respect to technical improvements, apart from robust software-engineering, we think it is also necessary to ensure appropriate patient identification (when dealing with thousands of participants) through advanced pattern matching methods which may rely on a combination of patient demographics and risk factors. This would ensure that there is no inadvertent misidentification of patients which may be harmful if treatment regimens are prescribed to the incorrect patient. Infrastructural barriers observed in our pilot study included power constraints as power supply was limited to fixed times of the day, and so the low-cost tablets had to be charged frequently or would not function optimally.

## Conclusions

In this study, we used an evidence-based CVD risk prediction and management tool to develop an mHealth platform in rural India for CVD screening and management with proper engagement of health care providers and local communities. Field evaluation found the platform to be acceptable and feasible for use. Detailed usage patterns could be gauged from analysing the interaction pattern of ASHAs with the mobile application. With over a third of screened participants being high risk, there is a need to demonstrate the clinical impact of the mHealth platform so that it could contribute to improved CVD detection in high risk low resource settings. With further technical improvements such as robust patient registration, we will conduct a two year cluster randomised trial covering 54 villages in southern India to evaluate the clinical impact of this mHealth platform.
